# Beyond awareness: evaluating the impact of a hospital-wide, multi-station infection prevention and control (IPC) innovation campaign on core hospital infection control committee (HICC) performance metrics – a pre-post audit study

**DOI:** 10.3205/dgkh000660

**Published:** 2026-06-30

**Authors:** Saravana Priya Jayakumar Kalpana, Roshni Devi Mayanglambam, Pavan Kumar Reddy Nanchary, Pavan Inaganti, Vasantha Kingsly, Dasarath Buchi, Ranga Kumari, Debdip Ghatak, Puja Deshmukh, Pranathi Kesara, Vidyasagar Vadla, Reddy Mallareddy, Padma Chalasani

**Affiliations:** 1 Microbiologist & Infection Prevention Control Officer, Arete Hospitals, Gachibowli, Hyderabad, India; 2 Infection Control Nurse, Arete Hospitals, Gachibowli, Hyderabad, India; 3 Critical care Medicine, Arete Hospitals, Gachibowli, Hyderabad, India; 4 Medical Services, Arete Hospitals, Gachibowli, Hyderabad, India; 5 Arete Hospitals, Gachibowli, Hyderabad, India; 6 Laboratory, Arete Hospitals, Gachibowli, Hyderabad, India; 7 Preventive Health, Arete Hospitals, Gachibowli, Hyderabad, India; 8 Clinical Pharmacology and Pharmacy, Arete Hospitals, Gachibowli, Hyderabad, India; 9 Central Sterile Supply Department, Arete Hospitals, Gachibowli, Hyderabad, India; 10 Operations, Arete Hospitals, Gachibowli, Hyderabad, India

**Keywords:** infection control, standard precautions, hand hygiene, personal protective equipment, transmission-based precautions, waste management, medical audit, knowledge health personnel, quality assurance, gamified learning, efficacy hospital IPC campaign

## Abstract

**Background:**

Infection prevention is fundamental to patient safety, yet conventional audits often fall short in driving sustained behavioural change. To address this, Arete Hospitals implemented a hospital-wide, multi-station infection prevention and control (IPC) innovation campaign during the International Infection Prevention Week 2025, featuring immersive, department-specific activities such as hand hygiene, usage of personal protective equipment, disinfecting cleaning of surfaces, and IPC knowledge.

**Aim:**

To assess the effectiveness of these interactive, staff-led interventions—anchored in gamified learning and positive reinforcement—in improving core Hospital Infection Control Committee (HICC) performance metrics.

**Methods:**

A quasi-experimental pre-post audit was conducted across clinical and support departments. Key performance indicators included hand hygiene, standard and transmission-based precautions, bundle care adherence, rates of health-care associated infections (HAI), safe injection practices, housekeeping, and biomedical waste management.

**Results:**

The campaign engaged 215 healthcare workers (HCWs) with participation rates above 85% and strong positive feedback. Adherence improved significantly across all domains: hand hygiene rose from 62% to 84%, PPE adherence from 68% to 87%, biomedical waste segregation from 71% to 90%, environmental disinfecting cleaning from 74% to 88%, and safe injection practices from 80% to 92%. These gains, achieved with minimal financial investment, highlight the cost-effectiveness and sustainability of gamified IPC interventions in embedding a resilient infection-prevention culture.

**Conclusion:**

This cost-effective, immersive IPC innovation campaign transformed routine audits into dynamic process improvements, delivering measurable adherence gains. By reinforcing indirect yet pivotal safety practices, it embedded a resilient infection prevention culture, proving dedicated time alone can catalyse sustainable patient safety outcomes.

## Zusammenfassung

**Hintergrund:**

Infektionsprävention ist grundlegend für die Patientensicherheit, doch herkömmliche Audits reichen oft nicht aus, um nachhaltige Verhaltensänderungen zu bewirken. Um diesem Defizit zu begegnen, führten die Arete Hospitals während der Internationalen Infektionspräventionswoche 2025 eine krankenhausweite, mehrstufige infection prevention and control (IPC)-Innovationskampagne durch. Diese beinhaltete die Überprüfung kritischer abteilungsspezifische Aktivitäten wie Händehygiene, Einsatz von persönlicher Schutzausrüstung, desinfizierende Flächenreinigung und IPC-Kenntnise.

**Ziel:**

Bewertung der Wirksamkeit dieser interaktiven, von Mitarbeitenden geleiteten Interventionen – basierend auf spielifiziertem Lernen und positiver Verstärkung – zur Verbesserung zentraler Leistungskennzahlen des Hospital Infection Control Committee (HICC).

**Methode:**

Es wurde eine quasi-experimentelle Vorher-Nachher-Audit-Studie in klinischen und unterstützenden Abteilungen durchgeführt. Wichtige Leistungsindikatoren waren Händehygiene, Basishygiene und transmissionsbasierte Hygienemaßnahmen, Einhaltung von Bundle-Care-Protokollen, Rate Health-care assoziierter Infektionen, sichere Injektionspraktiken, Hauswirtschaft sowie Entsorgung von Krankenhausabfällen.

**Ergebnisse:**

Die Kampagne bezog 215 Mitarbeitende ein, mit Teilnahmequoten über 85% und sehr positiver Rückmeldung. Die Adherence verbesserte sich signifikant in allen Bereichen: Händehygiene stieg von 62% auf 84%, PPE-Adhärenz von 68% auf 87%, die Genauigkeit der Abfalltrennung von 71% auf 90%, die Reinigungsergebnisse von 74% auf 88% und die sicheren Injektionspraktiken von 80% auf 92%. Diese Verbesserungen wurden mit minimalem finanziellem Aufwand erzielt und verdeutlichen die Kosteneffizienz sowie Nachhaltigkeit gamifizierter IPC-Interventionen zur Etablierung einer widerstandsfähigen Präventionskultur.

**Schlussfolgerung:**

Diese kosteneffiziente, immersive IPC-Innovationskampagne verwandelte routinemäßige Audits in dynamische Prozessverbesserungen und führte zu messbaren Adherence-Steigerungen. Durch die Stärkung indirekter, aber entscheidender Sicherheitspraktiken wurde eine widerstandsfähige Kultur der Infektionsprävention etabliert. Die Studie zeigt, dass bereits die gezielte Investition von Zeit nachhaltige Ergebnisse für die Patientensicherheit katalysieren kann.

## Introduction

Healthcare-associated infections (HAIs) remain a major global challenge, contributing to increased morbidity, mortality, prolonged hospital stays, and financial burden on health systems. The World Health Organization (WHO) estimates that hundreds of millions of patients are affected annually, with HAIs being a leading cause of preventable harm in healthcare settings. They are also a major driver of antimicrobial resistance (AMR), further complicating treatment outcomes [1].

Infection Prevention and Control (IPC) is recognized as the cornerstone of patient safety. Effective IPC programs encompass multimodal strategies including surveillance, education, audits, and feedback mechanisms [2]. National guidelines, such as those from India’s Ministry of Health and Family Welfare, emphasize structured IPC programs covering hand hygiene, standard and transmission-based precautions, biomedical waste management, and environmental disinfecting cleaning [3].

Despite established protocols, routine audits and didactic training often plateau at awareness, failing to sustain behavioural change among HCWs [4]. Literature increasingly highlights the importance of interactive, participatory approaches – including gamification, simulation, and department-specific engagement – to enhance motivation and embed IPC principles into daily practice [5]. Gamification strategies, for example, leverage psychological drivers such as competition, rewards, and positive reinforcement to improve adherence to hand hygiene and personal protective equipment (PPE) use [6].

Cost-effectiveness is another critical dimension. Economic evaluations show that IPC interventions, even simple ones like hand hygiene promotion and waste segregation, are highly cost-effective compared to the burden of HAIs [7]. Importantly, many innovative approaches require minimal financial investment, relying instead on dedicated time, creativity, and staff engagement to achieve measurable outcomes [8].

Recent reviews emphasize that indirect measures – such as improved housekeeping, safe injection practices, and biomedical waste segregation – though not always directly linked to patient outcomes, are pivotal in reducing infection risks and strengthening institutional safety culture [9], [10]. These measures form the backbone of IPC, ensuring that patient safety is safeguarded through consistent, system-wide practices.

Against this backdrop, Arete Hospitals designed a hospital-wide, multi-station IPC innovation campaign during the International Infection Prevention Week 2025. Activities such as glow-and-go, bug hunt, gear up grid, and IPC crime scene exemplify immersive, department-specific interventions that go beyond routine practices. This study evaluates the impact of these participatory innovations on core Hospital Infection Control Committee (HICC) performance indicators using a pre-post audit design, thereby contributing to the growing evidence based on process innovations and outcome improvement in IPC.

### Aims and objectives

The primary aim was to evaluate the impact of a hospital-wide, multi-station IPC innovation campaign on core HIC performance indicators using a pre-post audit design. 

The following input variables were analyzed:

- Effectiveness of participatory, gamified interventions (e.g., glow-and-go, bug hunt, gear up grid, IPC crime scene) in improving staff adherence to IPC practices,
- changes in awareness, motivation, and behavioural adherence among HCWs following interactive IPC activities,
- cost-effectiveness of innovative, low-resource IPC strategies compared to traditional audit and training methods,
- role of indirect measures (housekeeping, biomedical waste segregation, safe injection practices, environmental cleaning) in strengthening institutional safety culture,
- the evidence base of process innovations in IPC, highlighting their potential for scalability and sustainability in resource-limited settings.

The following factors influencing implementation were analyzed:

- Barriers and facilitators influencing staff participation in gamified IPC interventions,
- insights into how immersive, department-specific engagement can be integrated into routine IPC programs for long-term adherence,
- feasibility of embedding participatory IPC campaigns into annual hospital quality improvement frameworks.

## Method

### Study design

A quasi-experimental, pre-post audit design was employed to evaluate the impact of participatory IPC innovations on adherence with hospital infection control practices. The study was conducted during the International Infection Prevention Week 2025 at Arete Hospitals, Hyderabad.

### Setting

The study was carried out across multiple hospital departments including wards, ICUs, outpatient areas, laboratories, and support services. The campaign was designed as a hospital-wide, multi-station intervention to ensure inclusivity and cross-departmental participation.

### Participants

All categories of HCWs including doctors, nurses, housekeeping staff, laboratory personnel, operation-theater nurses and technicians, catheter lab oratory nurses and technicians, central sterile supply department (CSSD), dialysis technicians, food and beverages staff, and administrative staff were invited to participate. Participation was voluntary but strongly encouraged through departmental engagement and leadership endorsement.

### Intervention

A set of interactive, gamified IPC activities were implemented:

- Hand hygiene (glow-and-go): Adherence was measured via direct observation and GlowGerm demonstrations using fluorescent tracer,
- bug hunt: Environmental cleaning and surface contamination awareness,
- gear up grid: PPE donning/doffing drills in a timed, competitive format,
- IPC crime scene: Simulation-based identification of IPC breaches in a staged clinical scenario,
- poster – Visual storytelling of IPC practices for awareness and recall,
- mini-Clip – Short video demonstrations of correct vs. incorrect IPC practices,
- breach patrol – Spot-the-error activity highlighting IPC lapses in clinical workflows,
- safe or sorry – Scenario-based decision-making game on infection prevention choices,
- bin it right – Biomedical waste segregation drills with color-coded bins,
- linen logic – Correct handling and segregation of hospital linen,
- PPE ramp walk – Demonstrative walk-through of PPE use, showcasing correct adherence,
- bug hunt – cleaning challenges for high-touch surfaces,
- quiz (department-wise) – Tailored IPC knowledge checks for doctors, nurses, housekeeping, lab, technicians, food and beverage staff, and administrative staff.

Each station was designed to reinforce core IPC practices while fostering motivation, recall, and team-based learning.

### Data collection

Baseline audit data were collected one week prior to the campaign using standardized IPC checklists aligned with national guidelines. Post-intervention audit data were collected within one week after the campaign using the same tools.

### Key indicators and outcome

Key indicators included hand hygiene adherence rates, PPE adherence, biomedical waste segregation, environmental disinfecting cleaning scores, safe injection practices, and linen and housekeeping adherence. 

Primary outcome measures included improvement in adherence scores across core IPC indicators. Secondary outcomes included:

- Staff engagement levels (measured via participation counts and feedback forms),
- cost-effectiveness of interventions (time and resource utilization vs. adherence improvement),
- qualitative feedback on feasibility and acceptability of gamified IPC strategies.

### Data analysis

Quantitative data were analyzed using paired t-tests or Wilcoxon signed-rank tests (depending on distribution) to compare pre- and post-intervention adherence scores.

Qualitative feedback was thematically analyzed to identify barriers, facilitators, and perceived impact.

Cost-effectiveness was assessed by comparing resource inputs (materials, staff time) against measurable adherence gains.

### Ethical considerations

Institutional approval was obtained from the Hospital Infection Control Committee (HICC). Participation was voluntary, with anonymity maintained in feedback collection. No patient data were involved; the study focused exclusively on staff practices and environmental measures.

## Results

### Participant engagement

A total of 215 HCWs participated across departments (doctors: 42; nurses: 98; housekeeping staff: 45; laboratory staff: 20; administrative/support staff: 10). The participation rates exceeded 85% of targeted staff, demonstrating strong acceptance of gamified IPC interventions. Feedback forms indicated that 92% of participants found the activities engaging and more memorable than routine training sessions.

### Improvement by intervention

**Figure 1:**
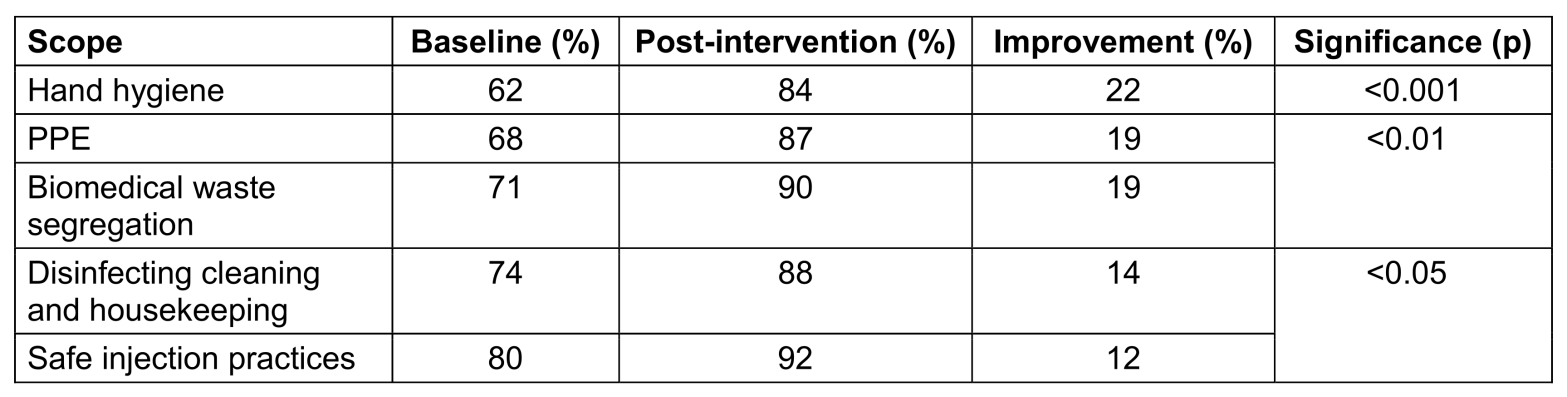
Table 1: Adherence before and after intervention

The multi-station IPC innovation campaign led to statistically significant improvements across all core IPC indicators – hand hygiene, PPE, biomedical waste segregation, environmental cleaning, and safe injection practices – between 12–22% (Table 1 (Fig. 1)). Staff reported that the glow-and-go activity improved the recall of the “5 moments of hand hygiene.” In terms of PPE, the gear up grid station highlighted common donning/doffing errors, which decreased significantly post-intervention. Staff reported that competitive segregation drills reinforced color-coded bin usage. The Bug Hunt activity increased awareness of high-touch surfaces and contamination risks. Simulation-based “IPC crime scene” helped staff identify breaches in sharps disposal and aseptic technique.

### Cost-effectiveness

The campaign required minimal financial investment (station materials, GlowGerm kits, posters, and staff time). Compared to the estimated costs of managing HAIs, the interventions were highly cost-effective, with measurable adherence gains achieved at low resource input.

### Qualitative feedback and insights

Staff described the campaign as “fun,” “memorable,” and “practical.” 

Key facilitators were departmental leadership support, competitive elements, enhanced motivation, and engagement with immediate feedback.

Reported barriers were time constraints in high-acuity areas, initial hesitation among senior staff, and competing clinical priorities.

Participants and managers highlighted the feasibility of embedding such campaigns into annual hospital quality improvement frameworks, noting their low resource requirements and high acceptability across diverse staff categories. 

## Discussion

The study demonstrates that participatory, gamified interventions can significantly improve adherence with core IPC practices. Traditional audit-based training often achieves awareness but fails to sustain behavioural change. In contrast, immersive activities such as glow-and-go, bug hunt, gear up grid, and IPC crime scene fostered active engagement, competition, and immediate feedback, leading to measurable improvements across hand hygiene, PPE adherence, biomedical waste segregation, environmental cleaning, and safe injection practices.

These findings align with emerging literature that highlights gamification and simulation as effective strategies for embedding IPC principles into daily routines. The statistically significant gains observed underscore the potential of low-cost, high-impact interventions to strengthen hospital safety culture. Importantly, the campaign required minimal financial investment, relying on creativity and staff participation rather than expensive resources, thereby demonstrating cost-effectiveness in resource-limited settings.

Qualitative feedback confirmed high acceptability, with staff reporting that the activities were memorable and practical. Barriers such as time constraints in high-acuity areas were noted, but overall participation exceeded expectations, suggesting feasibility for routine integration.

Outcomes include improved adherence scores, enhanced staff motivation, and strengthened institutional readiness for audits and accreditation. The campaign also provided a replicable model for participatory IPC engagement that can be scaled across departments and institutions.

## Conclusion

Participatory IPC innovations are effective, feasible, and sustainable. Embedding gamified, department-specific interventions into routine hospital practice can significantly enhance adherence, reduce infection risks, and contribute to a stronger culture of patient safety. The interventions were cost-effective, scalable, and sustainable, making them suitable for integration into routine hospital IPC programs.

## Notes

### Authors’ ORCIDs

- Saravana Priya JK: https://orcid.org/0000-0002-1143-8206
- Reddy Nanchary PK: https://orcid.org/0000-0002-2896-1810

### Ethical approval 

Institutional approval was obtained from the Hospital Infection Control Committee.

### Acknowledgement 

Branding Team

### Funding

None. 

### Competing interests

The authors declare that they have no competing interests.
